# Therapeutic Impact of Costus (*Saussurea lappa*) Against Ehrlich Solid Tumor-Induced Cardiac Toxicity and DNA Damage in Female Mice

**DOI:** 10.3389/fphar.2021.708785

**Published:** 2021-06-28

**Authors:** Rehab M. Elgharabawy, Ibrahim El Tantawy El Sayed, Nada Abd-Allah Rezk, Ehab Tousson

**Affiliations:** ^1^ Department of Pharmacology and Toxicology, Faculty of Pharmacy, Tanta University, Tanta, Egypt; ^2^ Department of Pharmacology and Toxicology, College of Pharmacy, Qassim University, Burydah, Saudi Arabia; ^3^ Chemistry Department, Faculty of Science, Menoufia University, Shibin El Kom, Egypt; ^4^ Zoology Department, Faculty of Science, Tanta, Egypt

**Keywords:** ehrlich solid tumor, costus root, cardiac functions, apoptosis, Ki67 expression

## Abstract

Breast cancer remains the most common cause of cancer deaths among women globally. Ehrlich solid tumor (EST) is a transplantable tumor model for simulating breast cancer. This study aims to describe the protective role of costus (*Saussurea lappa*) root against EST-induced cardiac toxicity. Forty female mice were randomly and equally divided into four groups (G1, control group; G2, costus group; G3, EST group; G4, EST + costus). The results showed that compared to the control, EST induced a significant increase in lactate dehydrogenase, creatine kinase, creatine kinase myoglobin, aspartate aminotransferase, and alkaline phosphatase activities; in potassium, chloride ion, cholesterol, triglyceride, and low density lipoprotein levels; in DNA damage and cardiac injury; and in p53 and vascular endothelial growth factor expression. Conversely, EST induced a significant decrease in sodium ion and high density lipoprotein levels and Ki67 expression compared to the control. Treatment of EST with costus improved cardiac toxicity, lipid profiles, electrolytes, and apoptosis, and protected against EST. This indicates the potential benefits of costus as an adjuvant in the prevention and treatment of cardiac toxicity.

## Introduction

Cardiac dysfunction is a sign of multi-factorial diseases, such as cancer and heart failure (Alotaibi et al., 2020; Alotaibi et al., 2021). Over 100 different types of cancer exist, each categorized by the type of cell initially affected. In many countries, the second most common cause of death after cardiovascular disease is cancer (Jemal et al., 2007). New cases of cancer are estimated to jump from 11.3 million in 2007 to 15.5 million in 2030, influenced in part by an increasing and aging global population (Wang et al., 2018; Ferlay et al., 2019).

Breast cancer remains the most common cause of cancer deaths among women worldwide, regardless of the emergence and evolution of new therapeutic approaches (Mutar et al., 2020). Ehrlich solid tumor (EST) is simulated breast cancer and has been used as a transplantable tumor model (Aldubayan et al., 2019; Tousson et al., 2020). The most important problem to solve in cancer treatment is the destruction of tumor cells without damaging surrounding natural cells. Recent studies have suggested that various anti-cancer agents act by inducing apoptosis in cancer cells (Abd Eldaim et al., 2021).

Medicinal herbs produce a wide range of chemical compounds that are important for treating various diseases, including cancer (Altwaijry et al., 2020). Costus (*Saussurea lappa*) is an aromatic perennial plant that is widely utilized in various indigenous medical systems globally for treating a variety of disorders, e.g., diarrhea, tenesmus, dyspepsia, vomiting, and inflammation (Tousson et al., 2019; Tousson et al., 2020). Costus is rich in antioxidants and has anti-hepatotoxic, anti-diabetic, antifungal, anthelmintic, anti-tumour, anti-inflammatory, anti-ulcer, antimicrobial, and immunostimulant effects (Nadda et al., 2020). Therefore, this study aimed to describe the protective role of costus root extract against EST-induced cardiac toxicity, injury, and alterations in apoptotic p53, pro-apoptotic Bax, and VEGF expression.

## Material and Methods

### Plant Material

Costus: Dry roots of *Saussurea lappa* were purchased from the local market of medicinal plant, Alexandria, Egypt. Root specimens were grounded into a fine powder.

### Determination of Total Phenolic Contents in Costus Roots Extracts

To determine the phenolic compounds from extract, the Folin-Ciocalteu method was used according to Mohsen and Ammar (2009).

### Determination of Total Flavonoids in Costus Roots Extracts

Total flavonoids content was determined using the aluminum chloride colorimetric method with quercetin as a standard and expressed (mg) as quercetin equivalent per gram of extract according to Zhishen et al. (1999).

### Determination of Total Antioxidant Capacity of *Costus* Roots Extracts

Total antioxidant activity was estimated by phosphomolybdenum assay according to Mohsen and Ammar (2009).

### Determination of DPPH Scavenging Activity of Costus Roots Extracts

The DPPH free radical solution is one of the most popular techniques for evaluation of radical scavenging activity, the method used that reported by (Brand-Williams et al., 1995) to evaluate the antioxidant activity spectrophotometrically.

### Experimental Animals

Experiments were performed on 60 female Swiss albino mice weighing 20–25 g obtained from the animal house colony of the Egypt Vaccine Company, Giza, Egypt. The animals were randomized and housed under ambient temperature (22–25°C) and relative humidity conditions, a 12 h light/12 h dark cycle, and two-week supply of a commercial diet and water ad libitum. Mice rearing and treatment throughout the experimental period were conducted in accordance with the Faculty of Science, Tanta University guide for animals, which was approved by the Institutional Animal Care and Use Committee (IACUC-SCI-TU-0047).

### Induction of Ehrlich Solid Tumor

The Egyptian National Cancer Institute (NCI; Cairo University, Egypt) provided the mice that carried Ehrlich ascites carcinoma (EAC). To maintain the tumor line and evaluate EST, viable cells (2.5–3 × 10^6^ cells/mouse) were implanted subcutaneously into the left thigh of each recipient mouse (Aldubayan et al., 2019).

### Experimental Design and Mice Groups

The mice were equally divided into four groups.

1^st^ group: Control group in which mice did not receive any treatment.

2^nd^ group: Costus group in which mice received costus extract (300 mg/kg body weight/day) orally *via* a stomach tube for 2 weeks (Abd Eldaim et al., 2019a).

3^rd^ group: EST group, including mice, each injected subcutaneously with 2.5–3 × 10^6^ EAC cells (Aldubayan et al., 2019).

4^th^ group: Post-treated EST with costus group, including mice, each first injected subcutaneously with 2.5–3 × 10^6^ EAC cells for 2 weeks to induce EST, before treatment with costus (300 mg/kg body weight/day) orally *via* a stomach tube for another 2 weeks.

### Blood Sampling

By the end of the experiment, mice were sacrificed *via* intraperitoneal injection of sodium pentobarbital, and subjected to complete necropsy. Blood samples were individually collected from the inferior vena cava of each mouse in non-heparinized glass tubes for estimation of cardiac function biomarkers and electrolytes.

### Cardiac Function Biomarkers

Serum lactate dehydrogenase (LDH) activity was measured by a kinetic method using kits (Vitro Scient, Cairo, Egypt) as described by Whitaker (1969). Serum creatine kinase (CK) levels were determined *via* an akinetic method using kits (Vitro Scient) as described by Zilva and Pannall (1985). Serum creatine kinase myoglobin (CK-MB) activity was determined using an assay kit (Bioassay Systems, Hayward, CA, United States ) as described by Bishop et al. (1971). Serum aspartate aminotransferase (AST) and alkaline phosphatase (ALP) activities were detected using a colorimetric method according to Moustafa et al. (2014) and Saggu et al. (2014), respectively.

### Electrolyte Estimation

Serum electrolyte (potassium, sodium, and chloride ions) levels were estimated using commercial kits (Sensa core electrolyte, India).

### Lipid Profile Estimation

Cholesterol, triglyceride, high-density lipoprotein-cholesterol (HDL-C) and low density lipoprotein-cholesterol (LDL-C) levels were determined using kits from ELLTECH.

### Comet Assay

One gram of crushed heart tissue was transferred to 1 ml ice-cold phosphate buffered saline (PBS) and the assay was performed according to Abd Eldaim et al. (2019b) for visualization of DNA damage.

### Histological Preparation

After necropsy the heart was immediately removed and fixed by immersion in 10% neutral buffered formalin solution for 24–48 h. The specimens were then dehydrated, cleared, and embedded in paraffin. Serial sections (5 µm thick) were sliced using a rotary microtome (Litz, Wetzlar; Germany) and stained with hematoxylin and eosin (Tousson, 2016). Histological changes scored on a 4-point scale: (−) none, (+) mild, (++) moderate, and (+++) severe damage.

### Immunohistochemical Detection for Apoptotic p53 Markers

Sections were incubated overnight at 4°C with anti-rabbit p53 monoclonal antibody to detect p53 expression (dilution 1:80; DAKO Japan Co., Ltd., Tokyo, Japan) according to Tousson et al. (2016).

### Immunohistochemical Detection of Vascular Endothelial Growth Factor Protein Expression in Heart Tissues.

The deparaffinized heart tissues sections were incubated overnight at 4°C with anti-rabbit VEGF monoclonal antibody (dilution 1:20; DAKO) to detect VEGF expression according to Youssef and Said (2014).

### Immunohistochemical Detection of Ki67

Ki67 immunoreactivity (Ki67-ir) was detected using avidin biotin complex (ABC) kits according to Al-Rasheed et al. (2018). The sections were incubated with anti-mouse Ki67 monoclonal antibody (dilution 1:50; DAKO) for 1–2 h at room temperature.

### Statistical Analysis

Data were expressed as mean values ± SD and statistical analysis was performed using one-way analysis of variance (ANOVA) followed by least significant difference (LSD) tests to assess significant differences among treatment groups. The criterion for statistical significance was set at *p* < 0.05. All statistical analyses were performed using SPSS statistical version 16 software package (SPSS^®^ Inc., United States ). The P53, VEGF and Ki67 positive cells were determined. Ten fields of vision selected randomly and the positive cells were scored (−) negative, (+) faint, (++) mild, (+++) moderate, and (++++) severe expressions according to Aldubayan et al. (2019).

## Results

### Total Phenolic and Total Flavonoid Contents in Costus Roots

The total phenols content in costus extracts showed the high amount of these compounds, with 3.502 mg/g of GAE, in contrast; total flavonoids contents, which are believed to be another determinant of the overall antioxidant activities, were measured, the quantities of flavanoids in the ethanolic extract of costus were found to be 6.377 mg CE/g.

### Total Antioxidant Activity in Costus Roots

#### DPPH

The ethanolic *costus* roots extract possessed radical-scavenging and antioxidant activities which figured out from the result 6.640 mg TE/g.

### Changes in Cardiac Function

Table 1 shows changes in cardiac functions in different groups under study. Compared to the control, significant increases were detected in CPK, LDH, CK-Mb, AST, and ALP levels in the EST group. Compared to the EST group, treatment of EST with costus (EST + costus) revealed significant decreases in CPK, LDH, CK-Mb, AST, and ALP levels.

**TABLE 1:** Effect of costus treatments on cardiac enzyme levels in the different groups studied.

	Control	Costus	EST	EST₊Costus
CPK (U/I)	295.8^#^ ± 6.24	278.4^#^ ± 8.13	892* ± 5.15	629.4^*#^ ± 8.96
LDH 10^3^ (U/I)	1.435^#^ ± 0.024	1.302^#^ ± 0.011	10.05* ± 0.27	7.814^#^* ± 0.23
CK-Mb (ug/ml)	0.268^#^ ± 0.008	0.227^#^ ± 0.003	0.364* ± 0.006	0.323^*#^ ± 0.008
ALP (U/I)	122.0^#^ ± 6.88	112.5^#^ ± 5.60	183.5* ± 9.54	151.8^*#^ ± 6.29
AST (U/I)	95.2^#^ ± 4.26	90.5^#^ ± 4.974	209.4* ± 8.39	148.5^*#^ ± 6.00

Data are expressed as mean ± SE of 10 observations. Where, EST, Ehrlich solid tumor; EST + Costus, treated Ehrlich solid tomor with Costus. Significant difference from the control group at *p < 0.05. Significant difference from the EST group at #*p* < 0.05.

### Changes in Electrolytes

Table 2 shows the changes in electrolytes in the different groups studied. A significant increase in k^+^ and Cl^−^ levels was detected in the EST group compared to the control. In contrast, Na^+^ level decreased significantly in the EST group compared to that in the control. Treatment of EST with costus resulted in significant decreases in k^+^ and Cl^−^ levels, and increase in Na^+^ level, compared to the EST group.

**TABLE 2:** Effect of costus treatments on electrolytes levels in the different groups studied.

	Control	Costus	EST	EST₊Costus
Na^+^ (mEq/I)	136.3^#^ ± 0.55	134.8^#^ ± 0.47	96.78^*^ ± 4.1	111.8^*#^ ± 1.1
K^+^ (mEq/I)	4.704 ± 0.109	4.566^#^ ± 0.10	6.612^*^ ± 0.074	6.358^*#^ ± 0.075
Cl^−^ (mEq/I)	101.1^#^ ± 0.64	101.0^#^ ± 0.51	120.1^*^ ± 1.1	107.7^#*^ ± 0.91

Data are expressed as mean ± SE of 10 observations. Where, EST, Ehrlich solid tumor; EST + Costus, treated Ehrlich solid tomor with Costus. Significant difference from the control group at *p < 0.05. Significant difference from the EST group at #*p* < 0.05.

### Changes in Lipid Profiles

Table 3 shows changes in the lipid profiles of the different groups studied. Significant increases in cholesterol, triglyceride, and LDL levels were detected in the EST group compared to the control. Conversely, HDL level decreased significantly in the EST group than in the control. Treatment of EST with costus resulted in significant decreases in cholesterol, triglyceride, and LDL levels, and increase in HDL level compared to the EST group.

**TABLE 3:** Effect of costus treatments on lipid profiles in the different groups studied.

	Control	Costus	EST	EST₊Costus
Cholesterol (mg/dl)	94.6^#^ ± 1.939	94.2^#^ ± 2.853	161.8* ± 2.478	132.8*^#^ ± 1.845
Tg (mg/dl)	105.6^#^ ± 3.108	97.4^#^ ± 2.943	166.0* ± 3.209	130.1*^#^ ± 1.761
HDL (mg/dl)	41.1^#^ ± 1.37	45.5^#^ ± 2.28	29.2* ± 1.02	32.0* ± 0.76
LDL (mg/dl)	31.38^#^ ± 3.018	29.34^#^ ± 4.045	99.4* ± 3.880	80.8* ± 1.966

Data are expressed as mean ± SE of 10 observations. Where, EST, Ehrlich solid tumor; EST + Costus, treated Ehrlich solid tomor with Costus. Significant difference from the control group at *p < 0.05. Significant difference from the EST group at #*p* < 0.05.

### DNA Fragmentation

**FIGURE 1:**
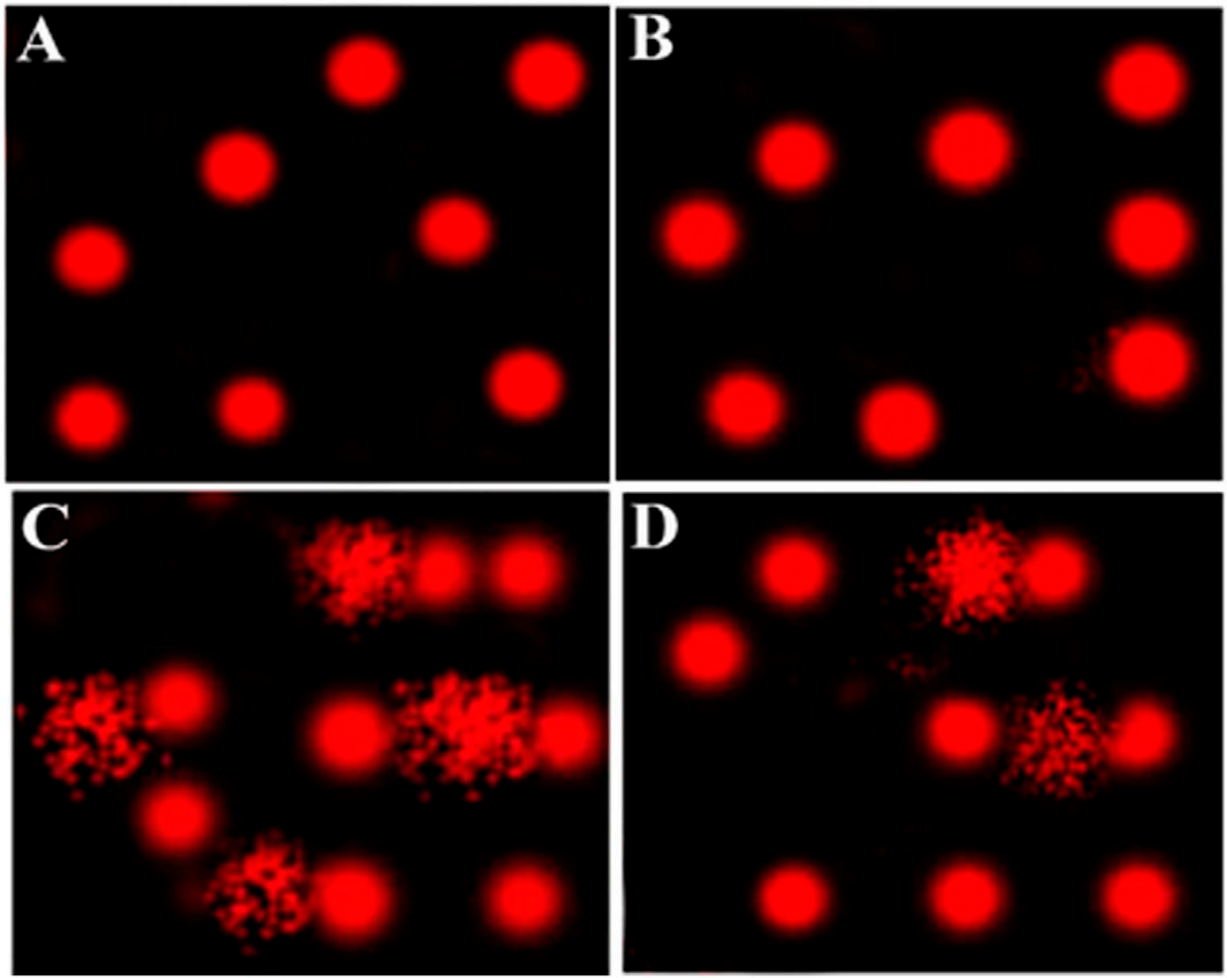
Comet assay for evaluation of heart tissue DNA damage. **(A**–**D)** Control, costus, EST, and EST + costus groups, respectively. EST, Ehrlich solid tumor.

A comet assay was performed to evaluate DNA damage in the heart of EST mice compared to the control. The results of the comet assay are shown in Table 4 and Figure 1. Administration of EST (G3) led to significant increase in DNA damage (*p* < 0.05), indicated by increased tail length, tail DNA%, and tail moment compared to the control (G1) and costus (G2) groups. This increased DNA damage was reduced after administration of costus in the post-treatment (G4) group for 2 weeks. Conversely, no significant difference in DNA damage (tail length) was observed between normal control and costus groups.

**TABLE 4:** Comet assay parameters obtained by image analysis in cells of all groups after treatment experiment.

	control	Costus	EST	EST₊Costus
Tailed%	2.0	3.0	25.5	12.0
Untailed%	98.0	97.0	74.5	88.0
Tails length µm	1.20^#^ ± 0.07	1.54^#^ ± 0.10	7.09*± 0.53	3.95^#*^±0.38
Tail DNA%	1.37	1.60	9.11	4.80
Tail moment	1.64	2.46	64.59	18.96

Data are expressed as mean ± SE of 5 observations. EST, Ehrlich solid tumor; EST + Costus, treated Ehrlich solid tomor with Costus. Significant difference from the control group at *p < 0.05. Significant difference from the EST group at #*p* < 0.05.

### Histological Changes in Heart

**FIGURE 2:**
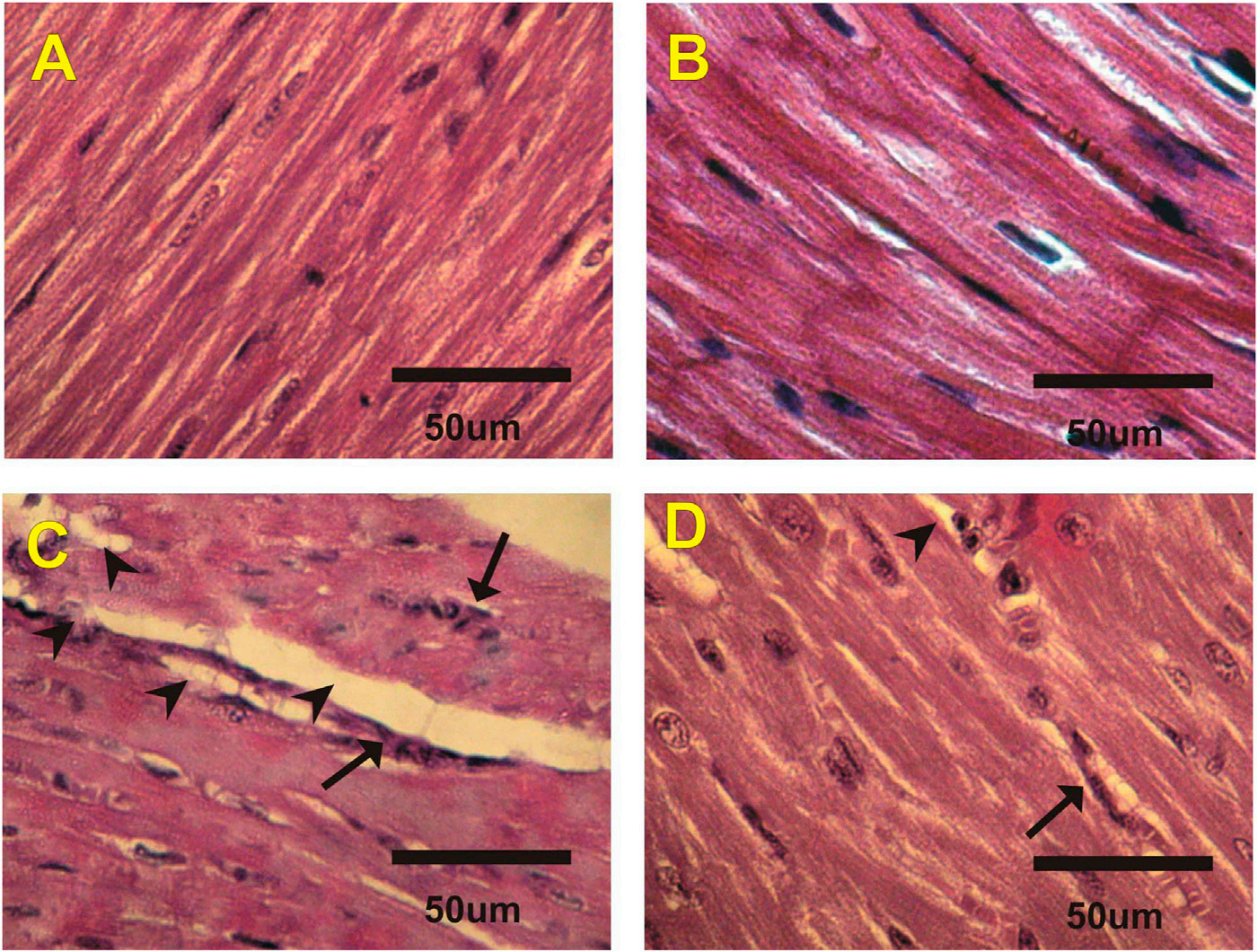
Photomicrographs of heart sections from different experimental groups stained with H&E. The **(A)** control (G1) and **(B)** costus (G2) groups show a normal myofibrillar structure with striations. **(C)** The EST group (G3) shows moderate myocardial atrophy and leukocyte infiltration (arrows). **(D)** The EST group treated with costus (G4) shows mild myocardial atrophy. H and E, hematoxylin and eosin; EST, Ehrlich solid tumor.

Figures 2A–D and Table 5 revealed the changes in mice heart histological structure in different groups. Light microscopy of heart sections from mice in the control and costus groups revealed a normal myofibrillar structure with striations (Figures 2A,B). Figure 2C shows marked hydrophobic changes in the myofibrillar structure with striations, moderate myocardial atrophy, nuclear pyknosis, and leukocyte infiltration in heart sections in the EST group. Conversely, heart sections in the EST + costus group revealed a normal myofibrillar structure with striations and only mild myocardial atrophy (Figure 2D).

**TABLE 5:** Effect of costus treatments on heart histological structure in the different groups studied.

	control	Costus	EST	EST₊Costus
Atrophy	−	−	+++	+
Leukocyte infiltration	−	−	+++	++
Nuclear pyknosis	−	−	++	+

Data are expressed as 10 fields of vision selected randomly and the changes were scored (−) negative, (+) faint, (++) mild, (+++) moderate, and (++++) severe. Where, EST, Ehrlich solid tumor; EST + Costus, treated Ehrlich solid tomor with Costus.

### p53 Immuohistochemical Changes in Heart

**FIGURE 3:**
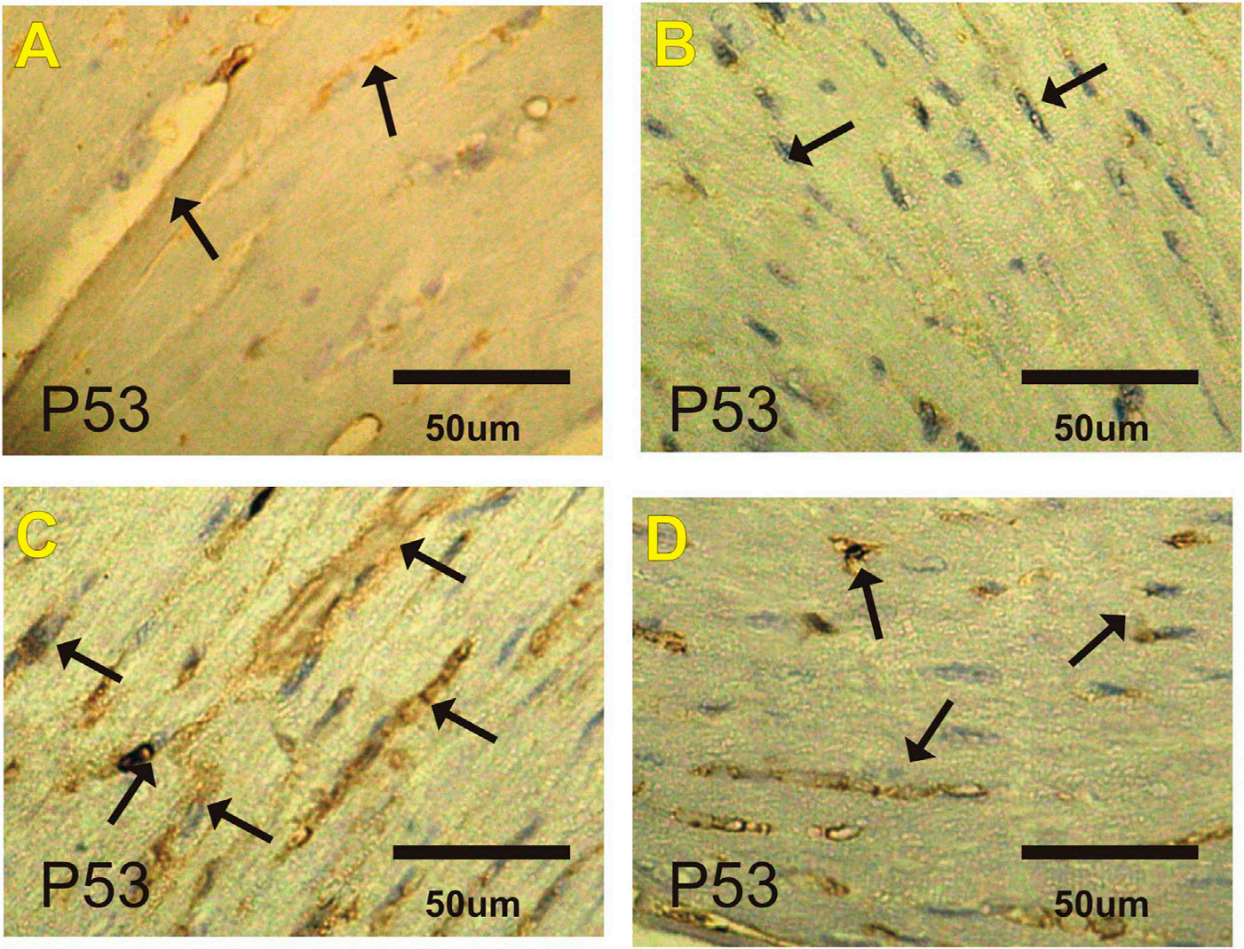
Photomicrographs of heart sections from the different experimental groups stained with p53-ir. Faint p53-ir reactions (arrows) are shown in the **(A)** control and **(B)** costus groups. **(C)** Severe positive reactions (arrows) for p53-ir are shown in the EST group. **(D)** Mild reactions (arrows) for p53-ir are shown in the EST group treated with costus. p53-ir, p53 immunoreactivity; EST, Ehrlich solid tumor.

Figures 3A–D and Table 6 show changes in mice heart sections stained for apoptotic p53 expression in different groups. Negative and faint positive reactions for p53 expression were observed in heart sections in the control and costus groups, respectively. In contrast, heart sections in the EST group showed severe positive reaction for p53 expression, while heart sections in the post-treated EST + costus group showed a mild reaction.

**TABLE 6:** Effect of costus treatments on P53, VEGF and Ki67 expressions on cardiac tissues in the different groups studied.

	control	Costus	EST	EST_+_Costus
P53	+	−	++++	++
VEGF	+	+	++++	+++
Ki67	++++	++++	++	+++

Data are expressed as 10 fields of vision selected randomly and the positive cells were scored (−) negative, (+) faint, (++) mild, (+++) moderate, and (++++) severe. Where, EST, Ehrlich solid tumor; EST + Costus, treated Ehrlich solid tomor with Costus.

### VEGF Immuohistochemical Changes in Heart

**FIGURE 4:**
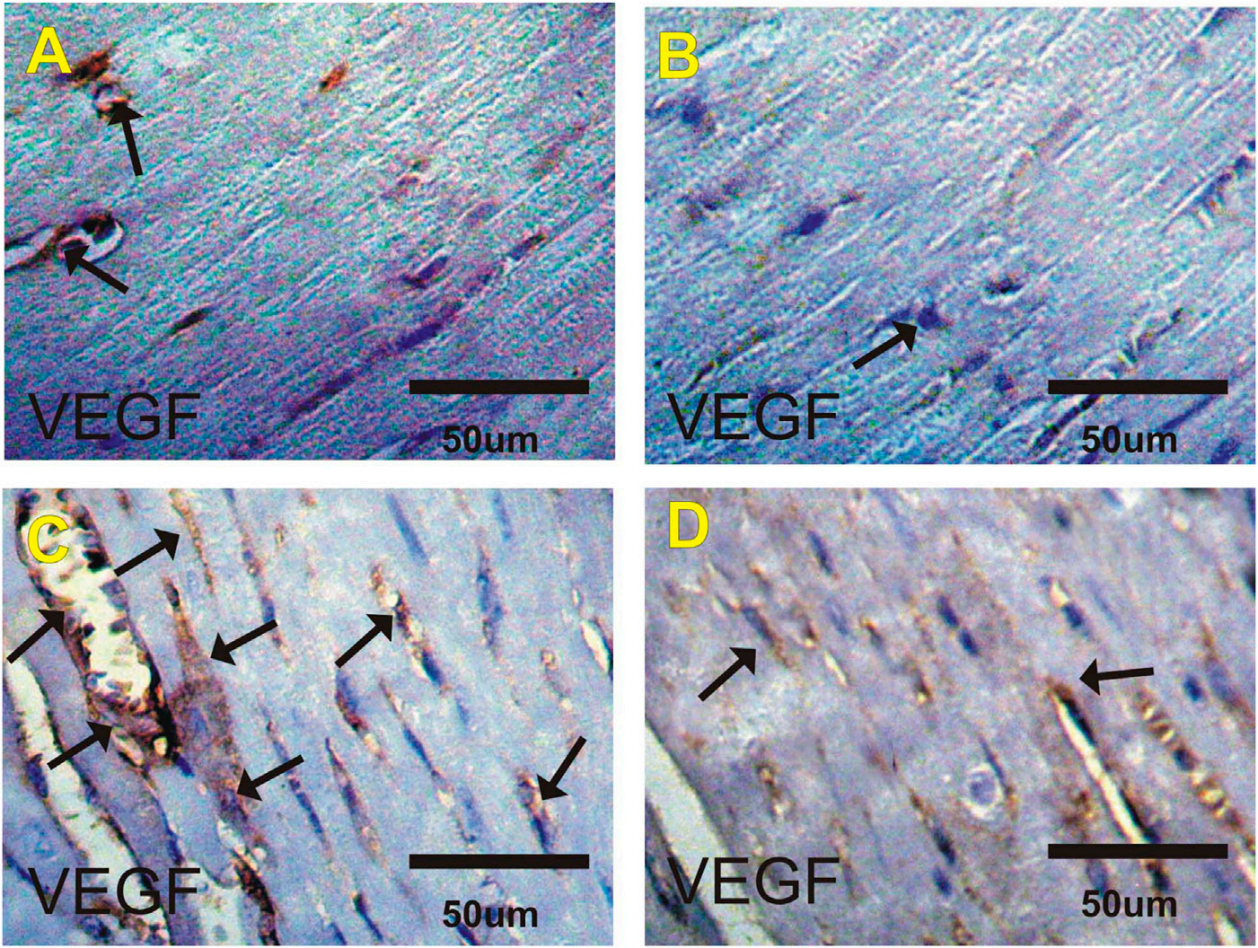
Photomicrographs of heart sections from the different experimental groups stained with VEGF-ir. Faint positive reactions for VEGF expression in the **(A)** control and **(B)** costus groups. **(C)** EST group shows moderate to severe positive reactions (arrows) for VEGF expression. **(D)** EST group treated with costus shows moderate reactions (arrows) for VEGF expression. VEGF-ir, vascular endothelial growth factor immunoreactivity; EST, Ehrlich solid tumor.

Figures 4A–D and Table 6 show changes in mice heart sections stained for VEGF expression in different groups. A faint positive reaction for VEGF expression was observed in cardiac sections in the control and costus groups respectively. Heart sections in the EST group revealed severe positive reaction for VEGF expression while the EST + costus group showed a moderate reaction.

### Ki67 Immuohistochemical Changes in Heart

**FIGURE 5:**
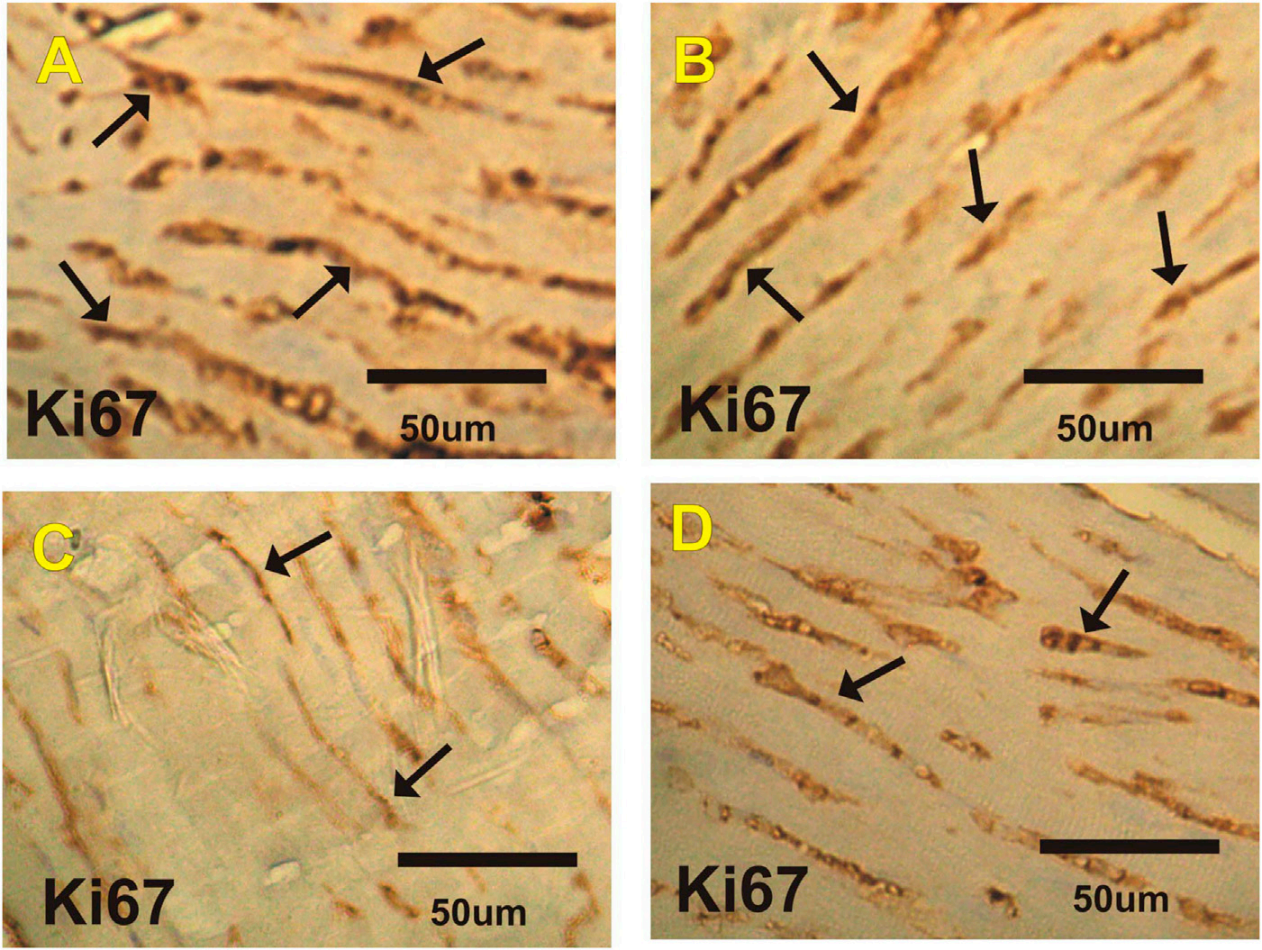
Photomicrographs of heart sections from the different experimental groups stained with Ki67-ir. Severe positive reactions (arrows) are shown for Ki67 expression in cardiac sections in the **(A)** control and **(B)** costus groups. **(C)** The EST group shows mild positive reactions for Ki67 expression. **(D)** The EST group treated with costus shows moderate reactions (arrows) for Ki67 expression. Ki67-ir, Ki67 immunoreactivity; EST, Ehrlich solid tumor.

Figures 5A–D and Table 6 show changes in mice heart sections from different groups stained for Ki67 expression. Severe positive reaction for Ki67 expression was observed in cardiac sections in the control and costus groups. Heart sections in the EST group showed a mild positive reaction for Ki67 expression, while the EST + costus showed a moderate reaction.

## Discussion

Cardiac dysfunction often occurs as the manifestation of multifactorial diseases, such as cancer (Murphy, 2016; Nguyen et al., 2016). No other human disease is more severe or associated with a greater mortality rate than cancer. The onset of cancer is triggered by cell DNA mutations resulting in the formation of an extraneous tissue mass known as a tumor (Wang et al., 2018). This current study aimed to describe the protective role of costus root extract against EST-induced cardiac toxicity.

This study showed that treatment with costus extract can decrease elevated EST-induced cardiac enzyme (LDH, CK, and CK-MB) levels. This result agrees with reports that EST (Aldubayan et al., 2019) and EAC (Noureldeen et al., 2017) induce increases in cardiac enzyme levels, and that methanol extract of costus has cardioprotective potential against carbon tetrachloride-induced cardiac toxicity (Njoku et al., 2017). In the current study, a significant increase in serum ALP and AST levels was observed, indicating liver and cardiac toxicity. The increase in liver enzymes may have arisen from a generalized destruction of hepatocytes and the release of AST into the plasma following tumor induction. These findings support previous reports (Abou Zaid et al., 2011; Aldubayan et al., 2019; El-Masry et al., 2020) that increased AST and ALP levels in EST can be interpreted as a consequence of liver and heart damage. In another study the protective effects of *Costus afer* leaf extract against CCl4-induced cardiotoxicity in rats were attributed to its antioxidant components (Njoku et al., 2017). Our study outcome is further consistent with the previous finding, that costus root aqueous extract modulates rat liver toxicity, and DNA damage, injury, and proliferation alterations induced by the plant growth regulator, Ethephon (Tousson et al., 2020).

Further, our study showed that EST significantly increased serum potassium and chloride ions levels, and significantly depleted sodium ions. Whereas, treatments with EST and costus decreased potassium and chloride ion levels, and increased sodium ion levels. These results agree with a report by Abd Eldaim et al. that EST-induced changes in kidney function and electrolytes, and was consistent with other reports that costus extract improved electrolyte levels against ethephon-induced kidney and testicular toxicity (Abd Eldaim et al., 2019a; Tousson et al., 2019).

The current results show a significant elevation in serum cholesterol, triglyceride, and LDL levels, but a significant decrease in HDL levels in EST mice compared to those in the control. Conversely, EST + costus treatment modulated reverse changes in lipid profiles. These results agree with Aldubayan et al. who reports that; EST decreases cholesterol and triglycerides levels (Aldubayan et al., 2019), and methanoic extracts of costus has hypolipidemic effects in diabetic rats (Jayasri et al., 2008; Eliza et al., 2009). Further, Anyasor et al. reported that costus treatments induced the depletion of the hematological and lipid profiles in arthritic rats after evaluation of a hexane fraction from the rat samples (Anyasor et al., 2014). These findings are parallel to those of Shediwah et al. who studied the antioxidant and antihyperlipidemic activity of *Costus speciosus* against atherogenic diet-induced hyperlipidemia in rabbits (Shediwah et al., 2019).

Hence, we suggest that EST induction leads to cardiac injuries by interfering with lipid metabolism. In addition to EST-induced changes in cardiac enzyme, electrolyte, cholesterol, and triglyceride levels, DNA damage may also induce myocardial infarction, and our study showed that cardiac injury sustained was ameliorated by EST + costus treatment. Our results agree with Aldubayan et al. who observed that EST induced cardiac injury, alteration in cardiac enzymes, and apoptosis (Aldubayan et al., 2019), but were parallel to reports by Mishra et al. that EAC induced cardiomyopathy in mice (Mishra et al., 2018). Inhibition of apoptosis is a vital previous step in tumor formation (Tousson et al., 2016). Our results show elevated p53 and VEGF expression and depleted Ki67 expression in heart tissues of EST mice compared to those in the control, indicating that EST induced apoptosis. Treatment of EST with costus modulated these alterations in p53, Ki67, and VEGF, consistent with a previous report Aldubayan et al. that EST induced elevation in p53 expression (Aldubayan et al., 2019). These findings indicate that EST induces cellular and molecular changes in mice hearts, which mimic changes in the hearts of patients with cancer. The inhibitory effect of costus on apoptosis may have played a role in the inhibition of the carcinoma and caused a reduction in their volumes.

## Conclusion

Summarily, our study showed that costus administration in EST-bearing mice improved cardiac function and structure and may kill the cancer cells through apoptosis, thereby regulating cancer cell proliferation and inhibiting its spread to other organs. Thus, costus may be used as a therapy that has been adapted to conform to human metabolic regulation, with the potential to constitute a natural chemotherapy.

## Data Availability

The original contributions presented in the study are included in the article/supplementary material, further inquiries can be directed to the corresponding author.

## References

[B1] Abd Eldaim M. A. Tousson E. El Sayed I. E. T. Abd Elmaksoud A. Z. Ahmed A. A. S. (2021). Ameliorative Effects of 9-diaminoacridine Derivative against Ehrlich Ascites Carcinoma-Induced Hepatorenal Injury in Mice. Environ. Sci. Pollut. Res. Int. 28, 21835–21850. 10.1007/s11356-020-11857-y 33415614

[B2] Abd Eldaim M. A. Tousson E. El Sayed I. E. T. Abd El-Aleim A. E.-A. H. Elsharkawy H. N. (2019b). Grape Seeds Proanthocyanidin Extract Ameliorates Ehrlich Solid Tumor Induced Renal Tissue and DNA Damage in Mice. Biomed. PharmacotherapyPharmacother 115, 108908. 10.1016/j.biopha.2019.108908 31108378

[B3] Abd Eldaim M. A. Tousson E. El Sayed I. E. T. Awd W. M. (2019a). Ameliorative Effects ofSaussurea Lapparoot Aqueous Extract against Ethephon-Induced Reproductive Toxicity in Male Rats. Environ. Toxicol. 34 (2), 150–159. 10.1002/tox.22669 30315693

[B4] Abou Zaid A. R. O. Hassanein M. R. R. El-Senosi Y. A. M. El- Shiekha F. (2011). Ameliorative Effect of Curcumin and Tannic Acid on Tumorinduced in Female Mice. Benha Vet. Med. J. 1, 61–69.

[B5] Al-Rasheed N. M. El-Masry T. A. Tousson E. Hassan H. M. Al-Ghadeer A. (2018). Hepatic Protective Effect of Grape Seed Proanthocyanidin Extract against Gleevec-Induced Apoptosis, Liver Injury and Ki67 Alterations in Rats. Braz. J. Pharm. Sci. 54 (2), 1–7. 10.1590/s2175-97902018000217391

[B6] Aldubayan M. A. Elgharabawy R. M. Ahmed A. S. Tousson E. (2019). Antineoplastic Activity and Curative Role of Avenanthramides against the Growth of Ehrlich Solid Tumors in Mice. Oxid. Med. Cell. Longev. 2019, 5162687. 10.1155/2019/5162687 30755785 PMC6348884

[B7] Alotaibi B. El-Masry T. A. Tousson E. Alarfaj S. J. Saleh A. (2020). Therapeutic Effect of Rocket Seeds (*Eruca Sativa* L.) against Hydroxyapatite Nanoparticles Injection Induced Cardiac Toxicity in Rats. Pak. J. Pharm. Sci. 33 (4), 1839–1845. 10.36721/PJPS.2021.34.1.SUP.337-343.1 33612468

[B8] Alotaibi B. Tousson E. El‐Masry T. A. Altwaijry N. Saleh A. (2021). Ehrlich Ascites Carcinoma as Model for Studying the Cardiac Protective Effects of Curcumin Nanoparticles against Cardiac Damage in Female Mice. Environ. Toxicol. 36 (1), 105–113. 10.1002/tox.23016 32865349

[B9] Altwaijry N. El‐Masry T. A. Alotaibi B. Tousson E. Saleh A. (2020). Therapeutic Effects of Rocket Seeds ( Eruca Sativa L .) against Testicular Toxicity and Oxidative Stress Caused by Silver Nanoparticles Injection in Rats. Environ. Toxicol. 35 (9), 952–960. 10.1002/tox.22931 32293792

[B10] Anyasor G. N. Funmilayo D. O. Odutola O. Olugbenga A. (2014). Hematological and Lipid Profile Evaluation of a Hexane Fraction of Costusafer Leaves in Arthritic Rats. Pharm. Biol. 53 (11), 1671–1676. 10.3109/13880209.2014.1001404 25857603

[B11] Bishop C. Chu T. M. Shihabi Z. K. (1971). Single Stable Reagent for Creatine Kinase Assay. Clin. Chem. 17, 548–550. 10.1093/clinchem/17.6.548 5574780

[B12] Brand-Williams W. Cuvelier M. E. Berset C. (1995). Use of a Free Radical Method to Evaluate Antioxidant Activity. LWT - Food Sci. Technology 28 (1), 25–30. 10.1016/s0023-6438(95)80008-5

[B13] El-Masry T. Al-Shaalan N. Tousson E. Buabeid M. Al-Ghadeer A. (2020). Potential Therapy of Vitamin B17 against Ehrlich Solid Tumor Induced Changes in Interferon Gamma, Nuclear Factor Kappa B, DNA Fragmentation, P53, Bcl2, Survivin, VEGF and TNF-α Expressions in Mice. Pak. J. Pharm. Sci. 33 (1), 393–401. 10.36721/PJPS.2020.33.1.SUP.393-401.1 32122873

[B14] Eliza J. Daisy P. Ignacimuthu S. Duraipandiyan V. (2009). Antidiabetic and Antilipidemic Effect of Eremanthin from Costus Speciosus (Koen.)Sm., in STZ-Induced Diabetic Rats. Chemico-Biological Interactions 182 (1), 67–72. 10.1016/j.cbi.2009.08.012 19695236

[B15] Ferlay J. Colombet M. Soerjomataram I. Mathers C. Parkin D. M. Piñeros M. (2019). Estimating the Global Cancer Incidence and Mortality in 2018: GLOBOCAN Sources and Methods. Int. J. Cancer 144 (8), 1941–1953. 10.1002/ijc.31937 30350310

[B16] Jayasri M. A. Gunasekaran S. Radha A. Mathew T. L. (2008). Anti-diabetic Effect of Costus Pictus Leaves in normal and Streptozotocin-Induced Diabetic Rats. Int. J. Diabetes Metab. 16 (3), 117–122.

[B17] Jemal A. Siegel R. Ward E. Murray T. Xu J. Thun M. J. (2007). Cancer Statistics, 2007. CA: A Cancer J. Clinicians 57 (1), 43–66. 10.3322/canjclin.57.1.43 17237035

[B18] Mishra S. Tamta A. K. Sarikhani M. Desingu P. A. Kizkekra S. Pandit A. (2018). Subcutaneous Ehrlich Ascites Carcinoma Mice Model for Studying Cancer-Induced Cardiomyopathy. Sci. Rep. 8 (1), 5599. 10.1038/s41598-018-23669-9 29618792 PMC5884778

[B19] Mohsen S. M. Ammar A. S. M. (2009). Total Phenolic Contents and Antioxidant Activity of Corn Tassel Extracts. Food Chem. 112 (3), 595–598. 10.1016/j.foodchem.2008.06.014

[B20] Moustafa A. H. A. Ali E. M. M. Moselhey S. S. Tousson E. El-Said K. S. (2014). Effect of Coriander on Thioacetamide-Induced Hepatotoxicity in Rats. Toxicol. Ind. Health. 30 (7), 621–629. 10.1177/0748233712462470 23042592

[B21] Murphy K. T. (2016). The Pathogenesis and Treatment of Cardiac Atrophy in Cancer Cachexia. Am. J. Physiol. Heart Circ. Physiol. 310, 466–477. 10.1152/ajpheart.00720.2015 26718971

[B22] Mutar T. F. Tousson E. Hafez E. Abo Gazia M. Salem S. B. (2020). Ameliorative Effects of Vitamin B17 on the Kidney against Ehrlich Ascites Carcinoma Induced Renal Toxicity in Mice. Environ. Toxicol. 35 (4), 528–537. 10.1002/tox.22888 31821727

[B23] Nadda R. K. Ali A. Goyal R. C. Khosla P. K. Goyal R. (2020). Aucklandia costus (Syn. Saussurea costus): Ethnopharmacology of an Endangered Medicinal Plant of the Himalayan Region. J. Ethnopharmacology 263, 113199. 10.1016/j.jep.2020.113199 32730877

[B24] Nguyen J. L. Yang W. Ito K. Matte T. D. Shaman J. Kinney P. L. (2016). Seasonal Influenza Infections and Cardiovascular Disease Mortality. JAMA Cardiol. 1 (3), 274–281. 10.1001/jamacardio.2016.0433 27438105 PMC5158013

[B25] Njoku U. O. Nwodo O. F. C. Ogugofor M. O. (2017). Cardioprotective Potential of Methanol Extract of Costus Afer Leaf on Carbon Tetrachloride-Induced Cardiotoxicity in Albino Rats. Ajprhc 9 (2), 51–58. 10.18311/ajprhc/2017/8363

[B26] Noureldeen A. F. Gashlan H. M. Qusti S. Y. Ramadan R. (2017). Antioxidant Activity and Histopathological Examination of Chromium and Cobalt Complexes of Bromobenz Aldehyde Iminacetophenone against Ehrlich Ascites Carcinoma Cells Induced in Mice. Int. J. Pharm. Phyto. Res. 7 (4), 7–12. 10.21276/bpr.2017.7.2.4

[B27] Saggu S. Sakeran M. I. Zidan N. Tousson E. Mohan A. Rehman H. (2014). Ameliorating Effect of Chicory (Chichorium Intybus L.) Fruit Extract against 4-Tert-Octylphenol Induced Liver Injury and Oxidative Stress in Male Rats. Food Chem. Toxicol. 72 (72), 138–146. 10.1016/j.fct.2014.06.029 25010453

[B28] Shediwah F. M. H. Naji K. M. Gumaih H. S. Alhadi F. A. Al-Hammami A. L. D'Souza M. R. (2019). Antioxidant and Antihyperlipidemic Activity of Costus Speciosus against Atherogenic Diet-Induced Hyperlipidemia in Rabbits. J. Integr. Med. 17 (3), 181–191. 10.1016/j.joim.2019.02.002 30799249

[B29] Tousson E. El‐Atrsh A. Mansour M. Abdallah A. (2019). Modulatory Effects of Saussurea Lappa Root Aqueous Extract against Ethephon‐induced Kidney Toxicity in Male Rats. Environ. Toxicol. 34 (12), 1277–1284. 10.1002/tox.22828 31392797

[B30] Tousson E. Hafez E. Abo Gazia M. M. Salem S. B. Mutar T. F. (2020). Hepatic Ameliorative Role of Vitamin B17 against Ehrlich Ascites Carcinoma-Induced Liver Toxicity. Environ. Sci. .Pollut. Res. 27, 9236–9246. 10.1007/s11356-019-06528-6 31916166

[B31] Tousson E. Hafez E. Zaki S. Gad A. (2016). The Cardioprotective Effects of L-Carnitine on Rat Cardiac Injury, Apoptosis, and Oxidative Stress Caused by Amethopterin. Environ. Sci. .Pollut. Res. 23 (20), 20600–20608. 10.1007/s11356-016-7220-1 27464663

[B32] Tousson E. (2016). Histopathological Alterations after a Growth Promoter Boldenone Injection in Rabbits. Toxicol. Ind. Health 32 (2), 299–305. 10.1177/0748233713500821 24097356

[B33] Wang X. Mo F.-M. Bo H. Xiao L. Chen G.-Y. Zeng P.-W. (2018). Upregulated Expression of Long Non-coding RNA, LINC00460, Suppresses Proliferation of Colorectal Cancer. J. Cancer 9 (16), 2834–2843. 10.7150/jca.26046 30123352 PMC6096368

[B34] Whitaker J. F. (1969). A General Colorimetric Procedure for the Estimation of Enzymes Which Are Linked to the NADH/NAD+ System. Clinica. Chim. Acta 24 (1), 23–37. 10.1016/0009-8981(69)90137-5 4305371

[B35] Youssef N. S. Said A. M. (2014). Immunohistochemical Expression of CD117 and Vascular Endothelial Growth Factor in Retinoblastoma: Possible Targets of New Therapies. Int. J. Clin. Exp. Pathol. 7 (9), 5725–5737. 25337214 PMC4203185

[B36] Zhishen J. Mengcheng T. Jianming W. (1999). The Determination of Flavonoid Contents in mulberry and Their Scavenging Effects on Superoxide Radicals. Food Chem. 64 (4), 555–559. 10.1016/s0308-8146(98)00102-2

[B37] Zilva J. F. Pannall P. R. (1985). Clinical Chemistry in Diagnosis and Treatment. London: Lloyd-Luke.

